# Expanding the Genetic and Phenotypic Spectrum of *POLRMT* ‐Related Mitochondrial Disease

**DOI:** 10.1111/cge.70011

**Published:** 2025-06-29

**Authors:** Mahmoud R. Fassad, Sebastian Valenzuela, Monika Oláhová, Jack J. Collier, Charlotte V. Y. Knowles, Eleni Mavraki, Miriam Elbracht, Nergis Güzel, Thomas Herberhold, Ingo Kurth, Andrea Maier, Larissa Mattern, Carol Saunders, Helen McCullagh, Katrin Õunap, Saskia B. Wortmann, Andre Reis, Lei Zhang, Claes M. Gustafsson, Robert McFarland, Robert W. Taylor

**Affiliations:** ^1^ Mitochondrial Research Group, Faculty of Medical Sciences, Translational and Clinical Research Institute Newcastle University Newcastle upon Tyne UK; ^2^ Human Genetics Department, Medical Research Institute Alexandria University Alexandria Egypt; ^3^ Department of Medical Biochemistry and Cell Biology University of Gothenburg Gothenburg Sweden; ^4^ Department of Applied Sciences, Faculty of Health & Life Sciences Northumbria University Newcastle upon Tyne UK; ^5^ Department of Neurology and Neurosurgery, Montreal Neurological Institute McGill University Montreal Quebec Canada; ^6^ NHS Highly Specialised Service for Rare Mitochondrial Disorders Newcastle upon Tyne Hospitals NHS Foundation Trust Newcastle upon Tyne UK; ^7^ Center for Human Genetics and Genomic Medicine, Medical Faculty RWTH Aachen University Aachen Germany; ^8^ Centre of Epilepsy for Children and Adolescents, Hospital for Neuropediatrics and Neurological Rehabilitation Schoen Klinik Vogtareuth Vogtareuth Germany; ^9^ Medical Treatment Center for Adults With Intellectual Disabilities and/or Severe Multiple Disabilities (MZEB) RWTH Aachen University Hospital Aachen Germany; ^10^ Department of Pathology and Laboratory Medicine Children's Mercy Hospital Kansas City Missouri USA; ^11^ Division of Clinical Genetics Children's Mercy Hospital Kansas City Missouri USA; ^12^ School of Medicine University of Missouri Kansas City Kansas City Missouri USA; ^13^ Department of Paediatric Neurology Leeds Teaching Hospitals NHS Trust Leeds UK; ^14^ Department of Clinical Genetics, Genetic and Personalized Medicine Clinic, Institute of Clinical Medicine, Tartu University Hospital University of Tartu Tartu Estonia; ^15^ University Children's Hospital Paracelsus Medical University (PMU) Salzburg Austria; ^16^ Institute of Human Genetics Friedrich‐Alexander‐Universität Erlangen‐Nürnberg (FAU) Erlangen Germany; ^17^ Department of Clinical Chemistry Sahlgrenska University Hospital Gothenburg Sweden

**Keywords:** mitochondrial disease, neurodevelopmental disorders, POLRMT, variant classification

## Abstract

Mitochondrial diseases are a complex group of conditions exhibiting significant phenotypic and genetic heterogeneity. Genomic testing is increasingly used as the first step in the diagnostic pathway for mitochondrial diseases. We used next‐generation sequencing followed by bioinformatic data analysis to identify potentially damaging variants in the *POLRMT* gene (NM_005035.4) in six new affected individuals. Structural protein analysis predicted the detrimental impact of variants on POLRMT protein structure. Patients show extended phenotypic abnormalities often presenting early in life with features including global developmental delay, cognitive impairment, short stature and muscular hypotonia. This study expands the genetic and phenotypic landscape of mitochondrial disease associated with *POLRMT* variants.

## Graphical abstract

We identified potentially damaging monoallelic and biallelic *POLRMT* variants in affected individuals from six unrelated families, thus extending both the clinical and genetic phenotypes of POLRMT‐related mitochondrial disease.

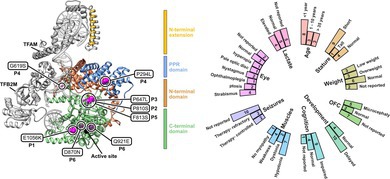

## 1 Introduction

The mitochondrial RNA polymerase (POLRMT), encoded by a nuclear gene, is responsible for the transcription of mtDNA along with the mitochondrial transcription factors A (TFAM) and B2 (TFB2M). It also plays a role in mtDNA replication by synthesising the RNA primers required for the initiation process [1, 2]. Previously, pathogenic *POLRMT*, monoallelic de novo and recessively inherited biallelic, variants were reported in patients presenting with developmental delay, intellectual disability, hypotonia and short stature. Structural modelling coupled with in vivo and in vitro assays confirmed defective mitochondrial transcription as the underlying disease mechanism [3].

Here, we report the clinical and genetic characteristics of six new patients from six unrelated families, thus expanding the clinical and genetic spectrum of POLRMT‐related mitochondrial disease.

## 2 Methods

Patients were recruited at six international clinical centres under locally established research ethics and governance regulations. Informed consent was obtained from all patients' parents or guardians. Patient recruitment, genetic testing and protein structural modelling are detailed in Suppporting Information Methods.

## 3 Results

### 3.1 Spectrum of *POLRMT* Variants

**FIGURE 1:**
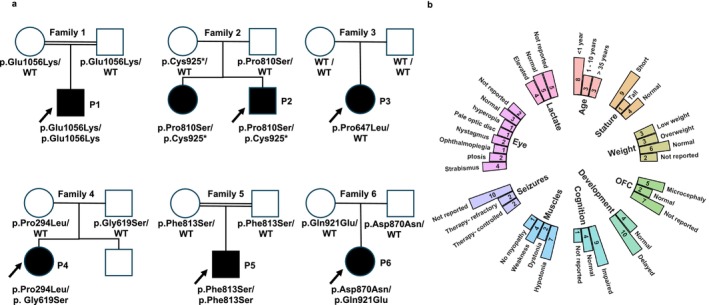
Family pedigrees, variant segregation and clinical characteristics of *POLRMT* patients. (a) Pedigrees of all 6 unrelated families reported in the current study show segregation of all reported variants within the available family members. (b) Clinical characteristics of patients with *POLRMT* variants reported in the current study and those previously reported by Oláhová et al. [3].

All patients harboured segregating, bi‐allelic variants with the exception of P3 who harbours a monoallelic de novo variant. (Figure 1a) Variants' nomenclature, allele frequency data and scores using in silico prediction tools are detailed in Suppporting Information Results and Table S1.

### 3.2 Clinical Features of *POLRMT* ‐ Related Mitochondrial Disease

Detailed clinical data of the patients reported in the current study are documented in Table 1 and Suppporting Information Results including the previously reported older affected sister in Family 2 [3]. Collective data including the current cohort of patients and those previously described (in total 14 patients from 12 unrelated families) are summarised in Figure 1b. Patients predominantly present with neurological features.

**TABLE 1:** Detailed clinical, molecular and biochemical data of patients with *POLRMT* variants.

Family	Family 1	Family 2		Family 3	Family 4	Family 5	Family 6
Patient	(P1)	Affected sister (previously reported)	(P2)	(P3)	(P4)	(P5)	(P6)
Variants	p.(Glu1056Lys)	p.(Pro810Ser); p.(Cys925*)		p.(Pro647Leu)	p.(Pro294Leu); p.(Gly619Ser)	p.(Phe813Ser)	p.(Asp870Asn); p.(Gln921Glu)
Inheritance	Recessive (homozygous)	Recessive (compound heterozygous)		De novo	Recessive (compound heterozygous)	Recessive (homozygous)	Recessive (compound heterozygous)
Gender	Male	Female	Male	Female	Female	Male	Female
Ethnicity	Kurdish	Caucasian		Caucasian	Caucasian	South Asian	Caucasian
Consanguinity	Yes	No		No	No	Yes	No
Age at first presentation	4 months	8 months	7 years	Birth	2 years	18 months	7 weeks
Age at genetic diagnosis	4 years	14 years	10 years	2 years	31 years	3 year 11 months	3 months
Growth	At age 2 year 7 months; height 85.5 cm (P2) weight 12 kg (P25) [subsequently on P91 at 8 year 6 months]; OFC 48.5 cm (P2)	Birth weight 2.69 kg (−1 SD), length 47 cm (−1 SD), OFC 32 cm (−1.5 SD) At 14 years; height 139 cm (−4 SD), weight 37.3 kg (−2 SD), OFC 49.5 cm (−3.5 SD)	Height 134 cm (−1 SD), weight 42.5 kg (+2 SD), BMI = 23.7 kg/m^2^ (> P95), OFC 52.5 cm (−1 SD)	NICU admission for 6 weeks for poor feeding and hypotonia; current height 106.9 cm (*Z* score: 0.78); weight 18.75 kg (*Z* score 0.85); BMI = 16.41	Normal growth till the age of 5 years, final height 147 cm (short stature), weight 52 kg, precocious puberty at the age of 6 years	Tall stature (P98) and OFC 49.5 cm at age 3 years and 11 months	Birth weight 3.63 kg, at 19 months of age; weight 8.4 kg (< P3), length 73 cm (< P3), OFC 42.5 cm (< P3)
Motor development	GDD with regression following stroke‐like episode at age 3 year 2 months	GDD	Normal motor development	Gross motor delay	ssssss	Normal motor development	Severe GDD
Dysmorphology	No apparent dysmorphic feature	Epicanthal folds, strabismus, upturned nose, opened mouth appearance, high palate, dysmorphic ears, bilateral simian creases	No apparent dysmorphic feature	No apparent dysmorphic feature, mild plagiocephaly	Secondary microcephaly, short, upturned nose, broad nasal tip, anteverted nares, scoliosis	Slightly large ears, broad mouth, and broad nasal tip	No apparent dysmorphic feature, high arched palate, scoliosis
Intellectual ability	Learning difficulties	Severe intellectual disability with no speech	Borderline intelligence	Normal intelligence	Moderate–severe intellectual disability, loss of expressive language	Predominantly speech delay/expressive language development disorder and regression in speech development	No eye contact, no fixation and only some sounds at 19 months
Behaviour	Aggressive outbursts with physical violence towards mother and siblings.	Irritability and aggressiveness	Physical and mental fatigue; difficulties in concentration	Normal	Alternation of days with hyperactivity and days with apathy	Diagnosis of autism spectrum disorder at the age of 3 years 3 months (repetitive/stereotypical movements, hand flapping, limited eye contact, sensitive to noise, outburst of rage, rarely contacting other children)	Too young to determine
Seizures	Focal onset with secondary generalisation, responding well to therapy	Focal seizures responded well to treatment	No	No	Therapy‐refractory seizures started at 2 years	No	Therapy‐refractory seizures started at 7 weeks
Eye	Pale optic discs	Strabismus	Hyperopia	Normal	Normal	Normal	Nystagmus
Muscles	Progressive dystonia, generalised dystonic spasms	Muscle hypotonia	Muscle weakness, muscle cramps	Hypotonia as infant	Progressive dystonia and truncal hypotonia	Muscle hypertonia early in life followed by hypotonia at 3 year 5 months	Generalized hypotonia
Sleep problems	No	Yes	No	No	Yes	Yes	No
Joints	Stiff but no fixed contractures	Club foot at birth	No	No	Secondary joint contractures	No	No
Gastro‐intestine	Gastrostomy feedings, constipation	Feeding difficulties in infancy	No	Dysphagia, Gastrostomy feedings, poor motility	Hypersalivation	No	Feeding difficulties
MRI brain	Loss of volume within the medial thalami and abnormal myelination, cerebellar atrophy and evidence of stroke‐like episode with occipito‐parietal infarct	Cavum septum pellucidum	Normal brain structures at the age of 10 years	Not done	Non‐specific deficit of white matter and corpus callosum	Not done	Polymicrogyria, corpus callosum agenesis, heterotopia, plexus cysts, unremarkable basal ganglia, unremarkable myelination
EEG	Generalised slowing with mixed theta/delta activity but no clear epileptiform discharges or electrographic seizures are noted	Unremarkable	Unremarkable	Not done	Diffuse, clearly bitemporo‐occipital left accentuated slow, high‐tension delta waves, partly occurring in rhythmic series.	Unremarkable	Not available
Muscle biopsy	Slight increase in fine pink granules in the cytoplasm on Gomori trichrome staining. 19% heteroplasmy for m.3242G>A	Not done	Not done	Not done	Type I fibre predominancy; biochemically normal pyruvate oxidation, rate ATP production and normal activities for the single enzymes of the oxidative phosphorylation system	Not done	Not done
Metabolic screening	Metabolic acidosis	Non‐specific organic aciduria Lactate/pyruvate ratio: 70 (normal range < 25)	Mildly increased alanine (670.2 μmol/L; normal 152–547); organic acids in serum and urine normal	Not done	Increased concentration of serum alanine	Not done	Not done
Serum lactate level	3.84–4.50 mmol/L	2.4 mmol/L	1.6 mmol/L	—	Normal	—	0.8 mmol/L

Abbreviations: EEG, electroencephalogram; GDD, global developmental delay; OFC; occipitofrontal circumference; SD, standard deviation.

### 3.3 Structural Analysis of POLRMT Variants

**FIGURE 2:**
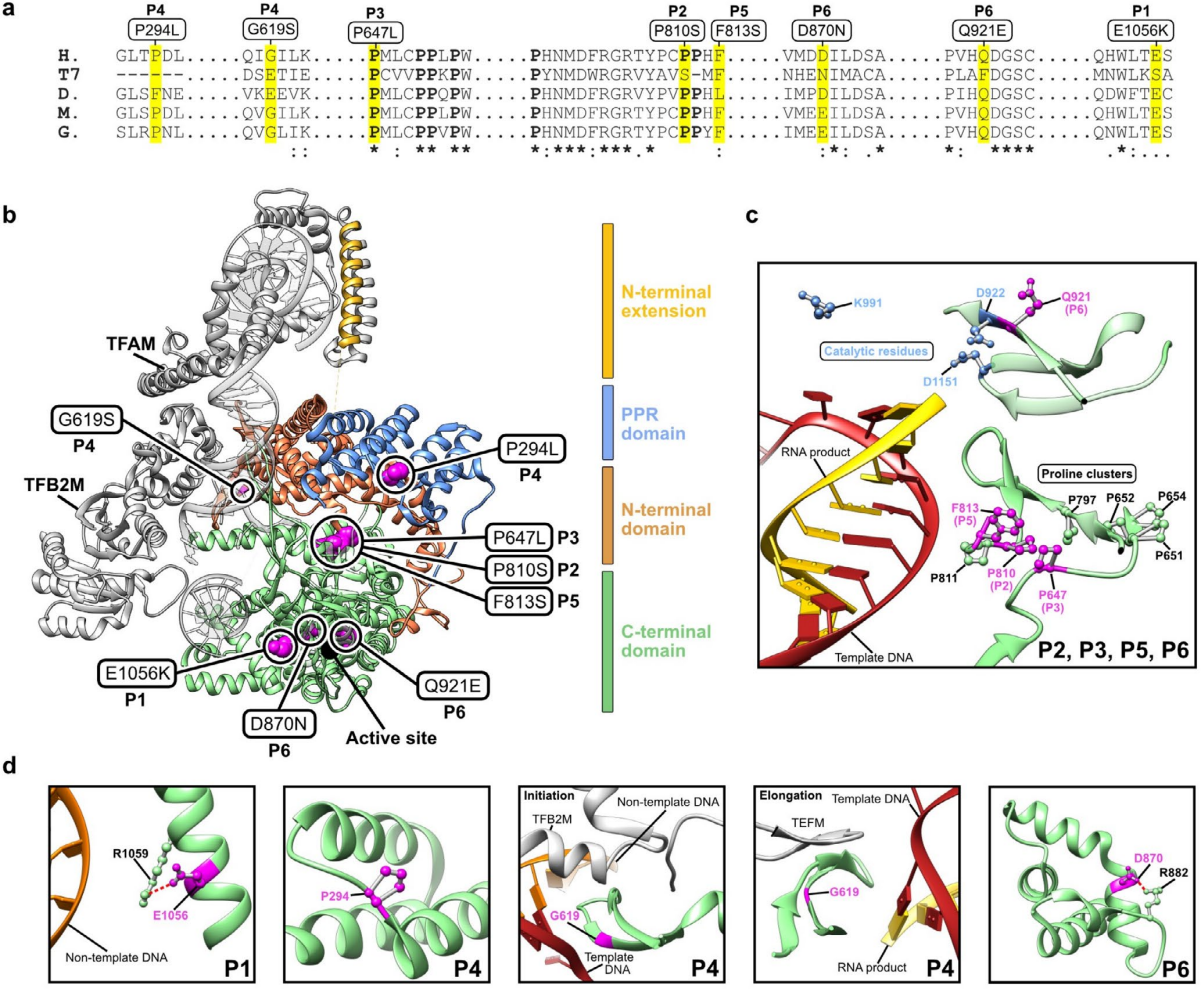
Sequence and structural analysis of mutations in POLRMT. (a) Multiple sequence alignment of POLRMT homologs; *Homo sapiens* (H.), *Bacteriophage T7* (T7), *Drosophila melanogaster* (D.), *Mus musculus* (M.) and *Gallus gallus* (G.). The locations of the mutations are highlighted in yellow, and the prolines in two strongly conserved proline clusters are marked with bold text. (b) Structure of the transcription initiation complex bound to the light‐strand promoter (LSP) (PDB ID: 6ERP). The four main domains of POLRMT are the *N*‐terminal extension (yellow), the pentatricopeptide repeat (PPR) domain (blue), the *N*‐terminal domain (orange) and the C‐terminal domain (green). The locations of disease‐causing variants are shown (magenta) and highlighted with black circles. The active site is indicated as a full black circle. TFAM, TFB2M and the DNA substrate is coloured grey. (c, d) Images were generated from the transcription elongation complex (PDB ID: 5OLA) and the initiation complex (PDB ID: 6ERP). Residues implicated in disease are coloured magenta and interactions are shown with dashed lines. (c) P2, P3 and P5 harbours substitutions of residues in the proline‐rich regions at the core of POLRMT, close to the DNA/RNA hybrid and the active site. P6 harbours a variant at the position adjacent to the catalytic residue D922. (d) P1 harbours a substitution of E1056, which forms a salt bridge with R1059, to a lysine. P4 harbours the P294L and the G619S variants. In addition to the Q921E variant, P6 also harbours the D870N variant. D870 forms a salt bridge with R882.

We performed a multiple sequence alignment of POLRMT homologs to determine if the residues implicated in disease were conserved across species. The analysis showed that all positions of interest are conserved or semi‐conserved across vertebrate and invertebrate species, while there are limited sequence similarities to the T7 RNA polymerase. However, two proline‐rich clusters in the centre of the enzyme are strongly conserved in all homologs (Figure 2a).

Next, we performed in silico analysis to predict if the identified variants could impact the structures of the mitochondrial transcription initiation or elongation complexes. The majority of the identified variants are located in the catalytic C‐terminal domain, with only two variants found in the other POLRMT domains (Figure 2b). Amino acid changes are described here using one letter code. The P647L (P3), P810S (P2) and F813S (P5) variants are all found in the conserved proline clusters, less than 6.0 Å from each other (Figure 2c). The unique conformational effect of prolines is likely essential to form the secondary structure in this region, which will be directly affected by the P647L (P3) and the P810S (P2) variants. The F813S (P5) variant will also impair the stability of this region as the bulky and hydrophobic phenylalanine sidechain is replaced with the small and hydrophilic serine sidechain. The Q921E variant (P6) is located close to the active site and the DNA/RNA hybrid during elongation (Figure 2c). As Q921 is adjacent to one of the catalytic residues (D922, K991 and K1151), it is likely that the Q921E variant will directly affect the catalytic activity of POLRMT. P1 harboursharbors the E1056K mutation, which disrupts a salt bridge normally formed between E1056 and R1059 (Figure 2d). This can potentially destabilize the region and leave R1059 free to interact with the DNA and hinder translocation during transcription. P4 harbours the P294L and G619S variants. The P294 proline is located between two helices in the PPR domain and acts as a helix breaker between the two. As leucine is often found in helices, the P294L mutation may impair the separation of the two helices and potentially cause the formation of one longer helix, thus altering the folding of the PPR domain (Figure 2d). The G619S variant is found on the intercalating hairpin, which is important for melting the DNA during initiation and removing the synthesized RNA during elongation (Figure 2d). The sequence of the intercalating hairpin is strongly conserved in vertebrates (Figure 2a), and the G619 residue may be important due to the unique flexibility provided by the glycine residue. In addition to the Q921E variant, P6 also harbors the D870N variant. D870 forms a stabilising salt bridge with R882, which is located on a nearby loop (Figure 2d). This salt bridge will be lost in the D870N variant and potentially replaced by a weaker hydrogen bond. Additionally, G619, P810, D870, Q921 and E1056 are all involved in hydrogen bonds with other residues, and mutations at these positions are likely to affect the stability of POLRMT.

## 4 Discussion

Mitochondrial diseases are highly genetically, biochemically and phenotypically heterogenous group of disorders. Even when clinical suspicion for mitochondrial disease is high, diagnostic confirmation has often been challenging. However, genomic analysis does offer an efficient and relatively inexpensive route to diagnose mitochondrial diseases [4, 5]. In the current study, we have used various NGS methods to identify new variants in *POLRMT* in six new patients, expanding the variant spectrum of *POLRMT*. Despite the wide phenotypic spectrum of *POLRMT*‐related mitochondrial disease, clinical symptoms of the newly reported patients confirm common features between affected individuals including predominant neurological manifestations such as motor developmental delay with impaired cognitive development affecting mainly speech and language and associated in several cases with behavioural problems. Although most patients presented in early life, a proportion of patients with *POLRMT*‐related pathology presented in adult life with different clinical problems (bilateral ptosis and ophthalmoplegia or muscle weakness) to those observed in the affected children. Short stature with or without microcephaly was reported in a large proportion of patients. Hypotonia, muscle weakness, seizures and several eye abnormalities were also among the frequent features of *POLRMT*‐related disease [3].

Both dominant and recessive patterns of inheritance were reported with no clear genotype/phenotype correlations. This may be explained by the variable impact of different variants such as loss‐of‐function, gain‐of‐function and dominant negative effect. Moreover, the variable extent of variants' damaging effect on the protein structure and/or function may also be another contributing factor [5]. Most variants were family specific. Only one variant p.(Asp870Asn) was reported in two different families in trans with a different pathogenic *POLRMT* variant in each of the patients (Family 6 in the current study and Family 1 previously reported by Oláhová et al. [3]). Both variants p.(Asp870Asn) and p.(Pro810Ser) are present in homozygous state in gnomAD, indicating potential hypomorphic impact of these variants. Considering high allele frequency and low in silico pathogenicity scores, these variants would have been classified as likely benign. However, protein structural analysis predicted them to destabilise protein structure. Consistently, they were shown to be associated with a moderate reduction in the in vitro mitochondrial transcription. Moreover, mild combined respiratory chain complex I and III deficiency was shown in the fibroblasts of the patient harbouring p.(Asp870Asn) variant [3]. This highlights the potential of protein structural analysis in predicting variant pathogenicity and the importance of adopting gene‐specific guidelines for variant interpretation.

Presentation and clinical symptoms of male and female siblings in Family 2 harbouring the same variants were distinct with the female patient presenting in very early childhood with severe manifestations of GDD, growth retardation and severe intellectual disability. Meanwhile, the male patient presented later in life at the age of 7 years with borderline intellectual disability and muscle weakness. However, his motor developmental milestones were acquired within normal time range. These findings raise the suspicion of potential sex‐specific effect in the clinical presentation of mitochondrial diseases [6, 7]. Interestingly, all the three patients presented with adult‐onset symptoms were male (including two siblings) [3].

MRI brain findings, when reported, are mostly non‐specific. Moreover, polymicrogyria, corpus callosum agenesis, heterotopia, and plexus cysts reported in P6 are unusual findings for mitochondrial disorder and may indicate other unidentified disease mechanisms besides the *POLRMT* mitochondrial pathology.

Protein structural analysis has demonstrated clinical utility and benefited classification of many missense variants in various genes [8]. It has proven to be a useful tool for prediction of the potential impact of variants in POLRMT protein structure. Consistent with previous reports, most variants described in the new patients were present in the C‐ terminal domain, with fewer variants in the N‐ terminal and PPR domains [3]. All variants are predicted to have detrimental impact on the structures of the mitochondrial transcription initiation or elongation complexes.

In summary, we have described the use of NGS coupled with protein structural analysis to identify apparently disease‐causing variants in *POLRMT* and shown several different clinical presentations associated with these variants, highlighting the wide clinical and genetic spectrum of *POLRMT*‐related mitochondrial disease.

## Author Contributions

Conceptualization: M.R.F., R.M. and R.W.T. Genetic and clinical investigations: M.R.F., M.O., J.J.C., C.V.Y.K., E.M., M.E., N.G., T.H., I.K., A.M., L.M., C.S., H.M., K.Õ., S.B.W., A.R., L.Z., R.M. and R.W.T. POLRMT protein structural analysis: S.V. and C.M.G. Writing of the first draft: M.R.F. Revision and editing of the manuscript: All authors.

## Conflicts of Interest

The authors declare no conflicts of interest.

## Supporting information


Data S1.

## Data Availability

The data that supports the findings of this study are available in Supporting Information Material of this article.
