# Supplementary material for: Expanding the Genetic and Phenotypic Spectrum of POLRMT ‐Related Mitochondrial Disease
Source: Clin Genet. 2025 Jun 29;109(1):167–75. doi: 10.1111/cge.70011 (PMC12674971; doi:10.1111/cge.70011)
Supplement: Supplementary file 1 — Data S1. [file CGE-109-167-s001.docx]

| **Family** | **Inheritance** | **Zygosity** | **gDNA (hg38)** | **cDNA**  **(NM_005035.4)** | **Protein** | **REVEL score** | **CADD**  **(38v1.6)** | **SIFT** | **PolyPhen2** | **AF in gnomAD v3.1.2** | **dbSNP** |
| --- | --- | --- | --- | --- | --- | --- | --- | --- | --- | --- | --- |
| 1 | Recessive | Homozygous | 19:619098C>T | c.3166G>A | p.(Glu1056Lys) | 0.074 | 22.7 | Deleterious (0.03) | Benign (0.271) | 1.98e-5 | rs948447720 |
| 2 | Recessive | Compound heterozygous | 19:621270G>A  19:620069G>T | c.2428C>T  c.2775C>A | p.(Pro810Ser)  p.(Cys925*) | 0.183  -- | 24  46 | Deleterious (0.04)  -- | Possibly damaging (0.450)  -- | 1.08e-3  3.28e-5 | rs201364510  rs55752843 |
| 3 | Dominant  (*de novo*) | heterozygous | 19:621758G>A | c.1940C>T | p.(Pro647Leu) | 0.929 | 25.4 | Deleterious (0.00) | Probably damaging (1.000) | -- | rs1984625887 |
| 4 | Recessive | Compound heterozygous | 19:625196G>A  19:621843C>T | c.881C>T  c.1855G>A | p.(Pro294Leu)  p.(Gly619Ser) | 0.251  0.396 | 21.9  27.7 | Deleterious (0.00)  Deleterious (0.02) | Benign (0.391)  Probably damaging (0.973) | 7.89e-5  -- | rs566027653  rs764240322 |
| 5 | Recessive | Homozygous | 19:621260A>G | c.2438T>C | p.(Phe813Ser) | 0.593 | 27.7 | Deleterious (0.01) | Possibly damaging (0.779) | -- | -- |
| 6 | Recessive | Compound heterozygous | 19:621090C>T  19:620367G>C | c.2608G>A  c.2761C>G | p.(Asp870Asn)  p.(Gln921Glu) | 0.133  0.518 | 14.2  24.2 | Tolerated (0.06)  Deleterious (0.00) | Benign (0.34)  Probably damaging (0.999) | 7.79e-3  5.26e-5 | rs139383492  rs749845654 |

**Table S1:** **Details of variants identified in the *POLRMT* gene**

**Supplementary Methods**

**Patient recruitment and genetic testing**

Patients were recruited at six international clinical centres under locally established research ethics and governance regulations. Informed consent was obtained from all patients’ parents or guardians. Self-reported consanguinity data were collected from all families. GeneMatcher facilitated collaboration between the recruiting centres (https://genematcher.org).

Parents of affected individuals at the time of examination did not report any symptoms suggesting mitochondrial disease. DNA samples of young children (typically less than two years old) presenting with unexplained global developmental delay (GDD), hypotonia and short stature were analysed using various next generation sequencing (NGS) modalities. In family 1, whole exome sequencing (WES) identified *POLRMT* variants in P1 and Sanger sequencing was used to segregate variants in the parents. In family 2, Sanger sequencing confirmed familial variants, previously identified in older affected sister by Trio-WES, in patient 2.^1^ Trio-WES was also performed in families 3, 4 and 6. Trio whole genome sequencing (WGS) was performed in family 5.

Bioinformatics data analysis and variants filtration and candidate prioritization were performed in the recruiting centres using standard procedures for rare diseases.^2^ In brief, variants were filtered based on their allele frequency in gnomAD and suspected mode of inheritance. Variants were further evaluated based on their impact at the protein level and the predicted pathogenicity scores using *in silico* tools including Polyphen-2, SIFT, Rare Exome Variant Ensemble Learner (REVEL) and Combined Annotation Dependent Depletion (CADD) scores.

**Structural modelling**

The structures of the POLRMT initiation complex (PDB ID: 6ERQ)^3^ and the elongation complex (PDB ID: 5OLA)^4^ were used to assess the structural implications of the patient variants. Analysis and image generation were performed in UCSF Chimera v.1.16.^5^ Multiple sequence alignment was performed with Clustal Omega.^6^ Sequences for the POLRMT homologs was obtained from the Uniprot database: *Homo sapiens* (O00411), *Bacteriophage T7* (P00573), *Drosophila melanogaster* (Q9VPW4), *Mus musculus* (Q8BKF1) and *Gallus gallus* (E1BZ13).^7^

**Supplementary results**

**Spectrum of *POLRMT* variants**

We identified six new patients from six unrelated families carrying rare *POLRMT* variants. (**Fig. 1a**) All patients harboured segregating, bi-allelic variants with the exception of P3 who harbours a monoallelic *de novo* variant. Variant nomenclature is based on the MANE Select *POLRMT* transcript (**NM_005035.4)** using current HGVS guidelines (<https://hgvs-nomenclature.org/>). The allele frequency of all variants was extracted from gnomAD database v3.1.2; CADD scores were calculated using CADD 38v1.6 (**Supplementary Table S1**).

All identified *POLRMT* variants were missense variants apart from the p.(Cys925*) nonsense variant in P2. Homozygous *POLRMT* variants were identified in two patients (P1 and P5), and three patients (P2, P4 and P6) harboured compound heterozygous variants. All variants were either absent or had low allele frequency and only present in the heterozygous state in gnomAD. One exception was the previously reported p.Asp870Asn variant found in P6, which was found in a homozygous state in 15 individuals in gnomAD.^1^ In trans to this variant, P6 also harbours c.2671C>G; p.(Gln921Glu) at the 3^rd^ bp upstream of splice donor site of exon 11 (SpliceAI score 0.01). The other exception was p.(Pro810Ser) variant in P2, which was also found in one homozygote in gnomAD. Variants’ nomenclature, allele frequency data and scores using *in silico* prediction tools are detailed in **Supplementary Table S1**.

The pathogenic mtDNA variant m.3242G>A (NC_012920.1) was found in heteroplasmy (19% in muscle, 32% in blood, 30% in buccal epithelium and 21% in urine in P1, while the mother had 4% heteroplasmy of the same variant in buccal epithelium and undetectable levels in urine and blood). At such low levels of heteroplasmy, it is very unlikely that this pathogenic mtDNA variant is contributing to the patient phenotype.^8^

**Clinical features of *POLRMT*- related mitochondrial disease**

Detailed clinical data of the patients reported in the current study are documented in **Table 1** including the previously reported older affected sister in family 2.^1^ Collective data including the current cohort of patients and those previously described (in total 14 patients from 12 unrelated families) are summarized in **Fig. 1b**. Patients predominantly present with neurological features.

In summary, over half of the patients (8/14 [57%]) presented in the first year of life. Intrafamilial variability was noted in family 2 as the older affected sister’s symptoms started at birth, meanwhile P2 presented at the age of 7 years. Oláhová M et al reported three patients who first presented in adult life.^1^ Although the concurrence of short stature and microcephaly was reported in 5/14 (35%) patients, short stature was also observed independently of microcephaly in 4 (28%) other patients. In contrast, another child was on the 98th centile for height at age 3 years and 11 months. Low birth weight +/- failure to thrive were reported in 3/14 patients (21%), overweight was reported in 3/14 patients (21%) and normal weight was reported in 6/14 patients (43%).

Most patients 9/14 (64%) showed motor developmental delay, frequently associated with impaired cognitive development especially for speech and language in 7/14 patients (50%). P3 presented with gross motor delay with normal intelligence. Borderline intellectual disability was noted in P2 who acquired motor developmental milestones at expected, age appropriate, timing’, meanwhile his older sister had GDD including both delayed motor development and intellectual disability with no speech development. P5 is another affected individual with normal motor development but with language development disorder, regression of linguistic skills and a diagnosis of autism spectrum disorder. Behavioral abnormalities were also noted including irritability, aggressive outbursts and hyperactivity. Sleep problems were reported in 3/14 patients (21%). Seizures were reported in 4/14 patients (29%), of which two had therapy-refractory seizures in P4 and P6. Muscle hypotonia was reported in 7/14 (50%), with generalized or proximal myopathy reported in 4/14 patients (29%). Dystonia was reported in 2/14 (14%). Ataxia was reported in only one patient (P4). Several ocular features were reported including strabismus in 4/14 patients (29%), ptosis in 2/14 patients, ophthalmoplegia in 1/14 patient, nystagmus in 2/14 patients, pale optic discs in 1/14 patient and hyperopia in 1/14 patient. In 3/14 patients, eye examination was unremarkable.

Several different brain MRI findings were reported. In P1, brain MRI showed loss of volume within the medial thalami, abnormal myelination, cerebellar atrophy and a resolved left occipito-parietal infarct. In family 2, MRI of the brain of the older sister showed cavum septum pellucidum while reported normal at the age of 10 years in the younger brother (P2). Nonspecific white matter loss and thinning of corpus callosum were reported in P4 as well as one of the previously reported patients.^1^ Brain MRI of P6 showed polymicrogyria, corpus callosum agenesis, heterotopia and plexus cysts, meanwhile the basal ganglia and myelination were unremarkable at the age of 3 months.

Few less frequent dysmorphic facial features were reported including large or dysmorphic ears were reported in 3/14 patients, upturned nose +/- broad nasal tip were reported in 3/14 patients. Moreover, high arched palate was also found in 2/14 patients.

Notably, cardiac abnormalities were not reported in any of the patients, except P3 who had bicuspid aortic valve, ventricular septal defect (VSD) and sinus venosus atrial septal defect (ASD). Sinus rhythm with a non-specific intraventricular conduction delay was found in her electrocardiogram.

**References**

1. Olahova M, Peter B, Szilagyi Z, et al. POLRMT mutations impair mitochondrial transcription causing neurological disease. *Nat Commun*. Feb 18 2021;12(1):1135. doi:10.1038/s41467-021-21279-0

2. Pedersen BS, Brown JM, Dashnow H, et al. Effective variant filtering and expected candidate variant yield in studies of rare human disease. *NPJ Genom Med*. Jul 15 2021;6(1):60. doi:10.1038/s41525-021-00227-3

3. Hillen HS, Morozov YI, Sarfallah A, Temiakov D, Cramer P. Structural Basis of Mitochondrial Transcription Initiation. *Cell*. Nov 16 2017;171(5):1072-1081 e10. doi:10.1016/j.cell.2017.10.036

4. Hillen HS, Parshin AV, Agaronyan K, et al. Mechanism of Transcription Anti-termination in Human Mitochondria. *Cell*. Nov 16 2017;171(5):1082-1093 e13. doi:10.1016/j.cell.2017.09.035

5. Pettersen EF, Goddard TD, Huang CC, et al. UCSF Chimera--a visualization system for exploratory research and analysis. *J Comput Chem*. Oct 2004;25(13):1605-12. doi:10.1002/jcc.20084

6. Sievers F, Wilm A, Dineen D, et al. Fast, scalable generation of high-quality protein multiple sequence alignments using Clustal Omega. *Mol Syst Biol*. Oct 11 2011;7:539. doi:10.1038/msb.2011.75

7. UniProt C. UniProt: the Universal Protein Knowledgebase in 2023. *Nucleic Acids Res*. Jan 6 2023;51(D1):D523-D531. doi:10.1093/nar/gkac1052

8. Wortmann SB, Champion MP, van den Heuvel L, et al. Mitochondrial DNA m.3242G > A mutation, an under diagnosed cause of hypertrophic cardiomyopathy and renal tubular dysfunction? *Eur J Med Genet*. Oct 2012;55(10):552-6. doi:10.1016/j.ejmg.2012.06.002
